# 2149. *In vitro* Activity of Aztreonam-avibactam and Comparator Agents Against Carbapenem-Resistant Enterobacterales With and Without Carbapenemases, ATLAS Global Surveillance Program, 2017–2021

**DOI:** 10.1093/ofid/ofad500.1772

**Published:** 2023-11-27

**Authors:** Mark G Wise, Francis Arhin, Daniel F Sahm

**Affiliations:** IHMA, Schaumburg, Illinois; Pfizer, Inc., Kirkland, Manitoba, Canada; IHMA, Schaumburg, Illinois

## Abstract

**Background:**

The investigational β-lactam/non-β-lactam β-lactamase inhibitor combination aztreonam-avibactam (ATM-AVI) is active *in vitro* against CRE isolates, including MBL-producers, as well as those co-producing serine β-lactamases of Class A, C, and some class D types. This study evaluated the *in vitro* activity of ATM-AVI and comparators against carbapenemase- and non-carbapenemase-producing CRE collected globally from 2017−2021 as part of the ATLAS surveillance program.

**Methods:**

85,990 non-duplicate, clinically relevant Enterobacterales isolates were collected from 258 medical centers located in 56 countries worldwide (excluding N. America and China). Susceptibility testing was performed by CLSI broth microdilution and interpreted using CLSI 2023 breakpoints. ATM-AVI was tested at a fixed concentration of 4 mg/L avibactam. A tentative ATM-AVI pharmacokinetic/pharmacodynamic (PK/PD) susceptible MIC breakpoint of ≤ 8 mg/L was applied for comparison purposes. Organisms with meropenem MIC >1 mg/L and *Escherichia coli*, *Klebsiella pneumoniae*, *Klebsiella oxytoca*, and *Proteus mirabilis* with aztreonam or ceftazidime MIC >1 mg/L were screened for β-lactamase genes by PCR and Sanger sequencing.

**Results:**

In total, 5,473 isolates were identified as CRE based on resistance to meropenem. ATM-AVI demonstrated potent *in vitro* activity against the CRE, with 99.3% of isolates (MIC_90_, 1 mg/L) inhibited at ≤8 mg/L (Table). ATM-AVI inhibited 99.5% of the carbapenemase-positive CRE, including 99.1% of the MBL-carriers (n=2,375) and 99.8% of serine-carbapenemase carriers (n=2,675) at ≤8 mg/L. Activity was somewhat reduced against the 441 CRE without detected carbapenemases as 96.3% (MIC_90_, 4 mg/L) of the isolates were inhibited at ≤8 mg/L aztreonam-avibactam. Amikacin was the most active comparator, although < 50% of each subgroup was susceptible at the CLSI breakpoint.

**Figure:**
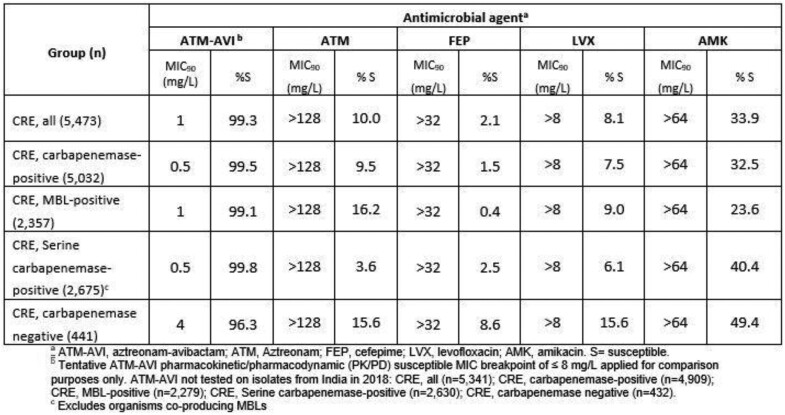

**Conclusion:**

Based on MIC_90_ values and the preliminary PK/PD breakpoint, ATM-AVI demonstrated potent *in vitro* activity against CRE, including MBL- and serine carbapenemase-carrying isolates. As limited therapeutic options presently exist for treating infections caused by CRE, further development of this agent appears warranted.

**Disclosures:**

**Mark G Wise, PhD**, Merck & Co., Inc.: Honoraria|Pfizer Inc.: Honoraria|Venatorx: Paid fees for conducting the study and abstract preparation **Daniel F. Sahm, PhD**, Merck & Co., Inc.: Honoraria|Pfizer Inc.: Honoraria|Venatorx: Paid fees for conducting the study and abstract preparation

